# Phytic Acid against *Clostridium perfringens* Type A: A Food Matrix Study

**DOI:** 10.3390/foods11030406

**Published:** 2022-01-30

**Authors:** Ana Paula Marinho Bloot, Daneysa Lahis Kalschne, Diego Ricardo Nunes Nogues, Joana S. Amaral, Eder Lisandro Moraes Flores, Eliane Colla, Sascha Habu, Ilton José Baraldi, Cristiane Canan

**Affiliations:** 1 Departamento Acadêmico de Alimentos, Universidade Tecnológica Federal do Paraná, Avenida Brasil, 4232, Bairro Independência, Medianeira 85884-000, Brazil; apmarinhobloot@gmail.com (A.P.M.B.); daneysa@hotmail.com (D.L.K.); nogues.diego@gmail.com (D.R.N.N.); ecolla@utfpr.edu.br (E.C.); baraldi@utfpr.edu.br (I.J.B.); canan@utfpr.edu.br (C.C.); 2 CIMO, Instituto Politecnico de Bragança, Campus de Santa Apolonia, 5300-399 Bragança, Portugal; 3 REQUIMTE-LAQV, Faculdade de Farmácia, Universidade do Porto, 4050-313 Porto, Portugal; 4 Departamento Acadêmico de Química, Universidade Tecnológica Federal do Paraná, Avenida Brasil, 4232, Bairro Independência, Medianeira 85884-000, Brazil; eder.utfpr@gmail.com; 5 Pró-Reitoria de Pós-Graduação, Universidade Tecnológica Federal do Paraná, Avenida Silva Jardim, 775, Curitiba 82590-300, Brazil; habu@utfpr.edu.br

**Keywords:** antimicrobial activity, inositol hexaphosphate, myo-inositol-1,2,3,4,5,6-hexakisphosphate

## Abstract

This study evaluated the inhibitory effect of phytic acid (PA) on the spore germination and vegetative cells growth of *Clostridium perfringens* type A, as well as its effect in combination with maltodextrin (MD) in cooked sausages. The addition of 1% PA showed a satisfactory inhibition of spores’ germination and vegetative cells growth of *C. perfringens* in BHI media. The inhibitory effect of 1% PA on vegetative cells was similar to the additive sodium sorbate (SS) at 10%. Subsequently, a mixture of PA-MD (1:1; *w/w*) was evaluated for the inhibition of *C. perfringens* spores in cooked sausages. The PA-MD 1.5% and 2.5% had a similar performance to SS 10% and a similar or higher performance than 0.015% NO_2_ (*p* < 0.05). In an unprecedented way, the present study demonstrated that PA inhibited spore germination and vegetative cells growth of *C. perfringens*, highlighting its potential use as an alternative and natural preservative for the meat industry.

## 1. Introduction

*Clostridium perfringens* is a spore-forming, anaerobic Gram-positive bacillus that is able to produce a large number of toxins. It is classified into five types (A-E) according to the production of four toxins, namely alpha, beta, epsilon, and iota, being also able to produce several other toxins such as the *Clostridium perfringens* enterotoxin (CPE), which is the main determinant of *Clostridium* type A food poisoning virulence [1,2]. *C. perfringens* is considered one of the most widely distributed pathogenic microorganisms in nature since their spores persist in soil and sediments and have a high prevalence in the gastrointestinal tract of humans and animals [3]. Therefore, it is frequently associated with foodborne illnesses and outbreaks. In the United States, *C. perfringens* was considered the third most common foodborne pathogenic bacteria causing confirmed, single-etiology outbreaks [4]. Also, in the United Kingdom, *C. perfringens* is the second most prevalent foodborne pathogen with estimates of around 80,000 gastroenteritis cases each year, being overpassed only by *Campylobacter* [5]. Food poisoning often occurs due to the consumption of foods that are contaminated with spores, particularly meat products that are cooked in large batches and slowly cooled or maintained at unsafe temperatures (10 to 54 °C), which allows spores germination and rapid microbiological growth [3,6]. CPE toxic effects are observed from 6 to 24 h after the ingestion of food that is contaminated with spores, causing relatively mild toxinosis [7]. This type of disease is generally characterized by watery diarrhoea and abdominal pain that last for less than 24 h, thus frequently resulting in under-reported cases.

*C. perfringens* vegetative cells are inactivated during the cooking processes. However, the spores are much more resistant, presenting reduction values at 100 °C that may range from 0.5 to 124 min, thus being likely to survive commercial cooking times and temperatures [8]. Moreover, the surviving spores may be heat-activated and subsequently germinate, outgrow, and multiply to hazardous levels, particularly if the products are maintained at inadequate temperature [9]. Also, spores are more resistant than their vegetative cells when facing treatments such as high hydrostatic pressure and the use of chemical preservatives [6,8]. As spores may survive hygienic-sanitary treatments and thermal processing, *C. perfringens* is a constant concern in the food industry [10]. Thus, alternative strategies that are suitable to promote spore inactivation and decrease the risk of *C. perfringens* food poisoning are still required [11]. Among those, different ingredients with antimicrobial activity or combinations thereof have been continuously investigated regarding their effectiveness against spore-forming bacteria. Those include low cost natural compounds such as organic acids and their salts (e.g., citric, sorbic and benzoic acids) [11], essential oils [12,13], and different bacteriocins such as pediocins [14,15], reuterin, and nisin [16,17] that showed satisfactory results against vegetative cell growth and spore germination.

Recently, several studies have suggested that phytic acid (PA) is effective in inhibiting the growth of different foodborne pathogens, mainly by the mechanism of cell membrane damage [18,19,20,21]. In particular, PA has been shown to have the capacity of inducing a great increase of the cell membrane permeability, leading to a decrease in the intracellular ATP concentration [18]. Inatsu et al. [22] described that a NaClO wash followed by a second wash with PA reduced *Escherichia coli* O157:H7 or *Listeria monocytogenes* in shredded cabbage or bean products, thus improving the sanitation of raw vegetables. PA and its combination with lysozyme significantly reduced *Pseudomonas* sp. in spoiled grass carp fillets [23], and the combination of PA and ethanol showed synergistic antimicrobial effects against *E. coli*, *Staphylococcus aureus*, *Bacillus subtilis*, and *Salmonella* Typhimurium [18]. Recently, it was [24] suggested that PA has promising potential applications in the food industry as a food preservative for shelf-life enhancement since it efficiently inhibited *S. aureus*, *L. monocytogenes,* and *S.* Typhimurium. Chemically described as myo-inositol-1,2,3,4,5,6-hexakisphosphate, PA has a great chelating potential due to its structure, with high affinity for polyvalent cations. Owing to its high capacity to bind iron, it acts as a potent inhibitor of iron-catalyzed formation of hydroxyl radicals, thus presenting a marked antioxidant activity [21,25,26,27]. PA antioxidant activity and potential antimicrobial activity render this compound a unique and versatile food preservative. In fact, even before it was recognized as “generally recognized as safe” (GRAS) by the Food and Drug Administration (FDA), PA was widely used as a food additive in many countries [25]. The GRAS notice 381 allows PA to be used as an antioxidant, chelating, and antibacterial agent in beverages, dairy products, processed vegetables, and vegetable juices [28].

Considering that PA and its metabolites are not toxic or reactive [21,29], its use as a food additive maybe an interesting alternative to other commonly used preservatives, particularly in meat products. In this context, the aim of this study was to evaluate the effects of PA against spores’ germination and vegetative cell growth of *C. perfringens* type A. Furthermore, the PA action as an antimicrobial agent was compared to conventional and currently used additives in cooked meat products, namely sodium sorbate and nitrate.

## 2. Materials and Methods

### 2.1. Bacterial Strains and Reactivation

The *C. perfringens* type A (INCQS: 00053, lot: 0788053, ATCC 3624) was supplied by the National Institute for Quality Control in Health (INCQS) of the Fundação Oswaldo Cruz (Rio de Janeiro, Brazil) in lyophilized form. The microorganism was kept under refrigeration until reactivation, which was performed by inoculating an aliquot of *C. perfringens* type A into 10 mL of Brain Heart Infusion (BHI) broth (Oxoid, Thermo Scientific, Hampshire, UK) and incubating anaerobically at 37 °C for 12 h.

### 2.2. Spore Preparation and Purification

Spores were obtained using a several steps approach as described by Paredes-Sabja et al. [30], with modifications. In the first step, an aliquot of 0.5 mL of BHI broth containing the reactivated strain was inoculated into 50 mL of BHI broth and incubated at 37 °C for 12 h. In the second step, 0.5 mL of the previously incubated BHI broth was inoculated into 10 mL of BHI broth and incubated at 37 °C for 8 h. In the third step, an aliquot of 0.5 mL of the BHI broth that was obtained in the second step was inoculated into 10 mL of Duncan-Strong spore medium [31] and incubated at 37 °C for 24 h. All incubations were conducted under anaerobic conditions. The sporulation was confirmed by optical microscopy, followed by spore purification through centrifugation of Duncan-Strong medium (2000 rpm for 10 min) (CT-5000R Centrifuge, Cientec, São Francisco, Brazil). The precipitate was washed using sterile distilled water and centrifuged for cell debris and germinated spores’ elimination. The spores that were obtained were resuspended in phosphate-buffered saline up to an optical density of 0.600 at 600 nm (OD_600_) (Lambda XLS, Perkin Elmer Spectrophotometer, Beaconsfield, UK), and stored at −20 °C until use.

### 2.3. Vegetative Cells Preparation

The vegetative cells of *C. perfringens* were obtained by inoculating an aliquot of 0.5 mL of the BHI broth containing the reactivated strain into 50 mL of BHI broth and incubated at 37 °C for 12 h. Subsequently, 0.5 mL of the incubated broth was inoculated into 10 mL of BHI broth that was incubated at 37 °C for 8 h and stored at −20 °C until use. All the incubations were conducted under anaerobic conditions.

### 2.4. Stock Solutions of the Tested Antimicrobials

The PA stock solution was prepared using rice dodecasodium phytate standard purity ≥ 90.00% (P8810, Sigma, St. Louis, MO, USA) and sterile distilled water. The stock solution of sodium sorbate (SS) (positive control) and sodium chloride (NaCl) (negative control) was also prepared using distilled water, and all the stock solutions were sterilised in an autoclave at 121 °C for 15 min and stored under refrigeration until use [11].

### 2.5. Clostridium perfringens Spore Germination in the Presence of Phytic Acid and Sodium Sorbate

The spores that were resuspended in phosphate-buffered saline (OD_600_ of 0.6) were activated by heating at 80 °C for 10 min and cooled at room temperature [30]. Subsequently, 1 mL of the activated spore suspension was inoculated into 50 mL of BHI broth containing PA at concentrations of 0.25%, 0.5%, 0.75%, and 1%. A positive control containing 10% SS, and a negative control containing only BHI broth and BHI with 0.90% NaCl were performed in parallel. The assays were incubated anaerobically at 37 °C for 24 h and spore germination was measured spectrophotometrically at 600 nm (OD_600_) in predefined intervals (0, 3, 6, 8, 9.5, 11, 12.5, and 14 h) [11]. All assays were performed in triplicate and the results were expressed as mean values of OD_600_.

### 2.6. Clostridium perfringens Vegetative Cells Growth in the Presence of Phytic Acid and Sodium Sorbate

The vegetative cells were thawed (12 h) and 0.1 mL was inoculated into 50 mL of BHI broth for activation, and subsequently incubated under anaerobic conditions at 37 °C for 3 h. After the incubation period, aliquots of 0.2 mL of the activated vegetative cells were inoculated in 10 mL of BHI broth containing PA (0.25%, 0.50%, 0.75%, and 1.00%), 10% SS, 0.90% NaCl, or only containing BHI. The assays were incubated anaerobically at 37 °C for 24 h and the growth was monitored by OD_600_ at 0, 4, 8, 12, and 24 h. Moreover, aliquots of all the tests were plated into BHI agar (Acumedia, Neogen, Lansing, MI, USA) using the pour-plate technique. The plates were incubated under anaerobic conditions at 37 °C for 24 h. All experiments of *C. perfringens* vegetative cells growth were performed in triplicate and the results were expressed as mean values of OD_600_ and as log CFU g^−1^.

### 2.7. Application of Phytic Acid in the Inhibition of C. perfringens Spores in Cooked Meat Products

The cooked sausage was selected as the meat product to be assayed for testing the *C. perfringens* spores’ inhibition potential because it is a food product that is widely consumed worldwide. The cooked sausage was prepared based on regular processing and formulation. The ingredients consisted of mechanically deboned poultry meat (40%), pork retail (25%), pork fat (15%), soy protein (4%), starch (2%), sodium chloride (1.5%), seasoning (0.5%), and ice (12%). The product was embedded in a plastic casing, cooked until reaching an internal temperature of 72 °C, peeled, and ground in a cutter for subsequent inoculation.

The spores were selected for inoculating the sausage based on preliminary results in which the spores showed a greater resistance to the application of the antimicrobials that were studied when compared to the vegetative cells.

The application of PA as an antimicrobial for meat products was achieved by mixing PA and maltodextrin (PA-MD) (1:1) in proportions of 1.5% and 2.5% (equivalent to 0.75% and 1.25% of PA, respectively; *w/w*). The 0.75% PA content was selected based on suitable results observed above 0.75% of PA in BHI tests, and the maximum PA percentage (1.25%) was employed to extrapolate the value of 1% of PA, which showed suitable results in the *C. perfringens* spores inhibition assay. Maltodextrin was used as a vehicle to facilitate industrial application. A sausage formulation containing 10% SS (*w/w*) [11] and another containing 150 μg g^−1^ of NO_2_ (0.015%; *w/w*) -the second is used by the meat industry as an additive for the inhibition of *Clostridium* sp.-were employed as a positive control. Additionally, the sausage formulation without antimicrobial addition was used as a negative control. The sausage formulations and respective codes are described below:

SA1: (negative control): only received the spores, in the same proportion as the others;

SA2: PA-MD (1:1) in the proportion of 1.5% (equivalent to a PA addition of 0.75%);

SA3: PA-MD (1:1) in the proportion of 2.5% (equivalent to a PA addition of 1.25%);

SA4: (positive control): SS in the proportion of 10%;

SA5: (positive control): NO_2_ in the proportion of 0.015% (150 μg g^−1^).

A sample of 310 g of each sausage formulation was inoculated with 6.2 mL (2%; *w/w*) of the purified spore solution (that was obtained as described in Section 2.2). Spore activation was performed by heating the solution at 80 °C for 10 min, followed by cooling at room temperature for 5 min before sausage application.

After inoculation, all the sausage formulations were anaerobically incubated at 37 °C for 57 h, and the development of *C. perfringens* was monitored at time intervals of 0, 3, 11, 18, 27, 42, and 57 h. A total of 10 g of each sausage formulation was resuspended in 0.1% peptone water (90 mL) and homogenized in a stomacher, serially diluted, and plated in sulphite polymyxin sulfadiazine agar (SPS) (Merck, Darmstadt, Germany) Petri dishes. The plates were incubated under anaerobic conditions at 37 °C for 24 h. All experiments were performed in triplicate and the results were expressed as the mean values in log colony forming units per gram (CFU g^−1^).

The experimental data of the growth curves were adjusted to the logistic model (LM) using predictive microbiology as previously described by Geitenes and Kalschne [32,33].

### 2.8. Statistical Analysis

All data were expressed as the mean ± standard error (*n* = 3) and analysed by ANOVA using the software Statistica 11.00 (Statsoft Inc., Tulsa, OK, USA), followed by Tukey’s test (*p <* 0.05). For the predictive microbiology data, the growth parameters lag phase extension (λ), exponential microbial growth rate (μ), and logarithmic increase in the population (*A*) were calculated by a nonlinear adjustment employing the same software. The suitability of the logistic model to the experimental growth curves was evaluated by mean square error (MSE), bias factor (BF), accuracy factor (AF), and coefficient of determination (R^2^) indices [33].

## 3. Results and Discussion

### 3.1. Germination of Clostridium perfringens Spores in the Presence of Phytic Acid and Sodium Sorbate

**Figure 1:**
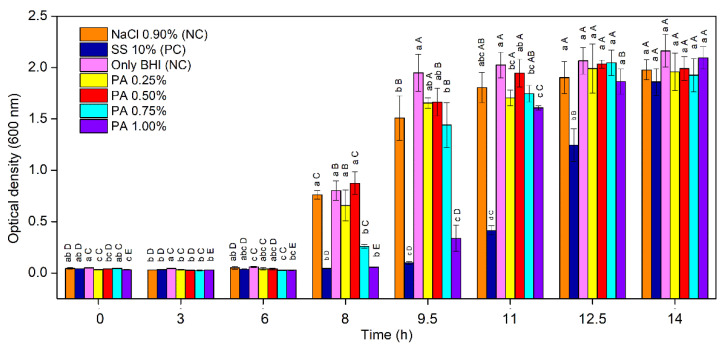
The effect of phytic acid (PA; 0%, 0.25%, 0.5%, 0.75%, and 1%), sodium sorbate (SS; 10%), and NaCl (0.9%) on the development of *C. perfringens* spores; PC: positive control; NC: negative control. The error bars represent the standard error (*n* = 3); different lowercase letters indicate statistically significant differences by Tukey’s test among samples at the same time (*p <* 0.05); different uppercase letters indicate statistically significant differences by Tukey’s test among the same sample at different times (*p <* 0.05).

The pH of the tested BHI broths with increasing PA were 7.71 (0.25%), 8.19 (0.5%), 8.53 (0.75%), and 8.80 (1%); the BHI broth without PA pH was 7.08; the pH was considered adequate for the development of *C. perfringens* and toxin production [34]. The effect of PA at different concentrations on the development of *C. perfringens* spores, as compared with only BHI, 10% SS and 0.9% NaCl, is shown in Figure 1, where absorbance is depicted as function of time.

It may be observed that during the first 6 h of incubation the spores of *C. perfringens* did not germinate (Figure 1). After 8 h of incubation, the spores’ germination was evident in the negative controls (only BHI and 0.9% NaCl) as well as in the media containing low PA concentration, namely 0.25% and 0.5% PA. For the media that was added with 0.75% PA, the spores’ germination was also observed, yet at lower values compared to the BHI broth and 0.9% NaCl. On the contrary, in the medium containing 1% PA and 10% SS, a clear inhibitory effect was observed, as the obtained result significantly differed from the previous intervals (*p* < 0.05). This effect was also maintained at 9.5 h of incubation since only slight spore germination, similarly with 10% SS, was observed (*p* > 0.05), attesting the 1.00% PA inhibition potential compared to lower PA concentrations and negative controls (*p* < 0.05). After 11 h of incubation, the inhibition that was caused by 1% PA was similar (*p* > 0.05) to the lower PA concentrations that were tested and similar to 0.9% NaCl. After 14 h, all assays reached the same level of spore germination, even in the positive control with 10% SS (*p* > 0.05).

*Clostridium perfringens* spores contain several different structural layers, which confer them resistance properties. The Gram-positive spore layer is composed of more than 50 specific proteins, providing protection against reactive chemicals and lytic enzymes [35]. *C. perfringens* spores may survive for a long time in the environment, however, under favourable conditions, they may germinate in time as short as 20 min. The results that were obtained in the current study for PA sporulation inhibitory effect in rich culture media was greater (more than six hours) when compared with the study of Alnoman et al. [11], who obtained satisfactory inhibitions up to three hours of incubation by adding sodium benzoate and potassium sorbate. While the antimicrobial activity of weak-acid preservatives, such as benzoic and sorbic acids, is attributed to undissociated acid, the PA effect may be attributed to its unique structure that ensures great chelating potential, forming a totally inactive chelate when binding to iron [25]. PA may chelate the iron of pyruvate ferredoxin oxidoreductase (PFO), one of the enzymes that is released by *C. perfringens* during spore germination [36]. This enzyme contains iron sulphur centres that are related to the oxidative cleavage system of pyruvate to acetate, which is the main route of adenosine triphosphate (ATP) synthesis, the production of H_2_, and electron transport in *Clostridium* species [37,38]. By inhibiting enzymatic activity, PA would interfere with the acetate production system, therefore interfering the spore germination phase due to the inhibition of ATP synthesis, with a consequent reduction in cell viability. The spore germination increased in the BHI broth containing 0.25%, 0.5%, and 0.75% PA after eight hours, as well as 1% PA after 9.5 h of incubation, may be associated with a reduction in the PA content that is available in the medium at non-inhibitory levels, which would allow the germination of dormant spores [39].

### 3.2. Clostridium perfringens Vegetative Cells in the Presence of Phytic Acid and Sodium Sorbate

**Figure 2:**
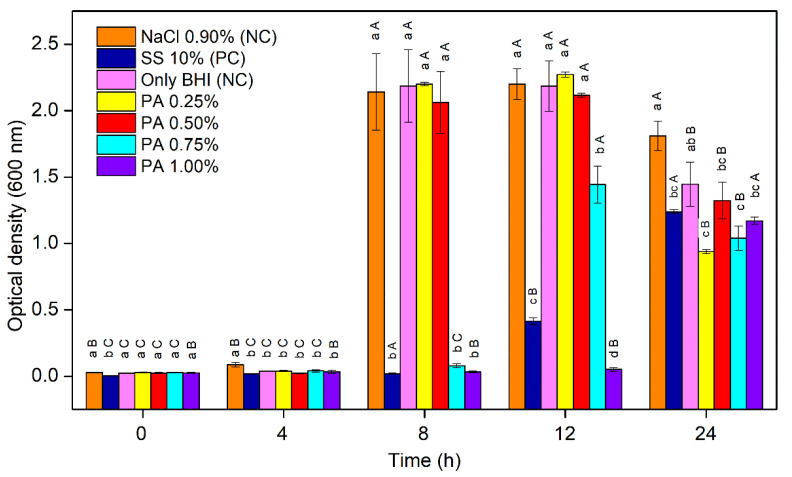
The effect of phytic acid (PA; 0%, 0.25%, 0.5%, 0.75%, and 1%), sodium sorbate (SS; 10%), and NaCl (0.9%) on the growth of *C. perfringens* vegetative cells; PC: positive control; NC: negative control. The error bars represent the standard error (*n* = 3); different lowercase letters indicate statistically significant differences by Tukey’s test among samples at the same time (*p <* 0.05); different uppercase letters indicate statistically significant differences by Tukey’s test among the same sample at different times (*p <* 0.05).

Figure 2 shows the inhibition of *C. perfringens* vegetative cells by PA at different concentrations and compares it with the only BHI, 10% SS, and 0.9% NaCl.

After up to 4 h of incubation, no growth of the vegetative cells was observed in the assays, with the exception of the only BHI and 0.9% NaCl that presented a slight, but statistically significant, increase of OD_600_. At 8 h of incubation, both the negative control and assays with 0.25% and 0.5% PA presented OD_600_ values that were significantly higher than 10% SS, 0.75% PA, and 1% PA (*p* < 0.05). After 12 h of incubation, only 10% SS and 1% PA assays had an inhibitory effect on vegetative cells growth (*p* < 0.05); moreover, comparing 10% SS and 1% PA, the latter had a greater inhibitory effect (*p* < 0.05). After 24 h of incubation, the inhibition that was promoted by 10% SS and 1.00% PA decreased significantly, with a similar increase in OD_600_ for both (*p* > 0.05). In the other assays, an OD_600_ reduction was observed compared with data from 12 h, which could be attributed to a biomass precipitation.

**Figure 3:**
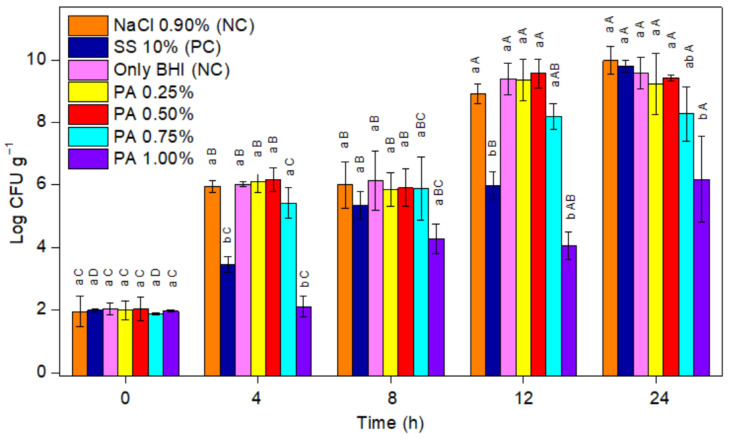
The effect of phytic acid (PA; 0%, 0.25%, 0.5%, 0.75%, and 1%), sodium sorbate (SS; 10%), and NaCl (0.9%) on the growth of *C. perfringens* vegetative cells (CFU mL^−1^); PC: positive control; NC: negative control. The error bars represent the standard error (*n* = 3); different lowercase letters indicate statistically significant differences by Tukey’s test among samples at the same time (*p <* 0.05); different uppercase letters indicate statistically significant differences by Tukey’s test among the same sample at different times (*p <* 0.05).

The PA inhibitory effect at different concentrations on *C. perfringens* cells was also evaluated by the plate count and expressed in log CFU mL^−1^ (Figure 3). In general, the obtained results are in agreement with those that were obtained by OD_600_ increase. The number of viable colonies that were found was similar (*p* > 0.05) among the negative controls (BHI and BHI with 0.90% NaCl) and assays with lower PA contents (0.25%, 0.5%, and 0.75% PA) for the 24 h evaluated, demonstrating a negligible antimicrobial effect of PA in concentrations < 0.75%. On the other hand, an inhibitory effect was shown by assays with 10% SS and 1% PA, both presenting a similar number of viable colonies at all incubation times up to 12 h (*p* > 0.05) that was significantly lower (*p* < 0.05) compared to the negative controls. However, at 24 h, a stronger antimicrobial effect that was attributed to 1% PA was evident as the number of viable colonies was up to three log cycles lower than 10% SS (*p* < 0.05).

As previously described for spores, the inhibitory effect of PA on *C. perfringens* vegetative cell growth may be related to its chelating action on the enzymes ferredoxin and PFO. Both ferredoxin and PFO contain iron sulphur centres and are closely related to the oxidative cleavage of pyruvate to acetate, and, therefore, to the synthesis of adenosine triphosphate (ATP), H_2_, and to the transport of electrons in *Clostridium* metabolism [37,38]. Due PA pronounced chelating potential, it could chelate the iron molecule from ferredoxin and PFO, blocking its enzymatic action and interfering with acetate production, thus reducing cell viability due to the inhibition of ATP synthesis. In fact, PFO has already been suggested as a potential target for drug design against certain anaerobes [40]. The inhibition of the enzymatic activity of ferredoxin and pyruvate ferredoxin oxidoreductase may also interfere with most metabolic pathways of *Clostridium* oxidation, including the metabolism of sulphur, purines, hydrogen, and nitrogen [41].

### 3.3. Application of Phytic Acid in the Inhibition of C. perfringens Spores in Cooked Meat Products

**Figure 4:**
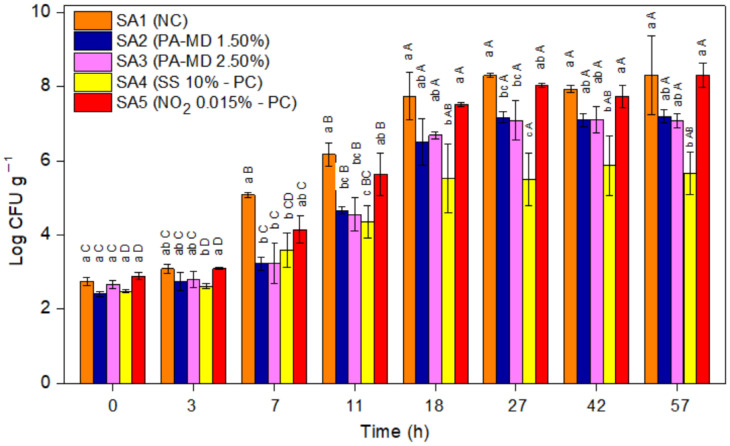
The effect of phytic acid-maltodextrin (PA-MD; 1.50% and 2.50%), sodium sorbate (SS; 10%), and sodium nitrite (NO_2_; 0.015%) on *C. perfringens* spore development in cooked sausage (SA); PC: positive control; NC: negative control. The error bars represent the standard error (*n* = 3); different lowercase letters indicate statistically significant differences by Tukey’s test among samples at the same time (*p <* 0.05); different uppercase letters indicate statistically significant differences by Tukey’s test among the same sample at different times (*p <* 0.05).

The PA effect in meat products was compared to nitrate because, similar to other countries, in Brazil, only sodium or potassium nitrate and sodium or potassium nitrite are allowed as food preservatives in cooked meat products, with a concentration of 0.03% and 0.015% (*w/w*) respectively [42]. Considering a previous study [11], the sodium sorbate was tested at a concentration that was 200 times higher than allowed in some countries for cured meat products (500 μg g^−1^) [43]. The inhibition of *C. perfringens* spore germination in cooked sausage is presented in Figure 4. Between 0 and 3 h of incubation, there was no difference in the development of *C. perfringens* among all the sausage formulations (*p* > 0.05). After 7 h, the sausages with the added PA-MD 1.50% and 2.50%, SS 10%, and NO_2_ 0.015% showed *C. perfringens* spores germination that was lower than the negative control (*p* < 0.05). These results are in accordance with those that were previously reported by Alnoman et al. [11]; the authors evaluated the growth of *C. perfringens* spores in cooked meat that was added with 5% SS and verified a reduction of more than 1 log cycle in viable cells. After 11 h, the formulations of the added PA-MD at both concentrations and 10% SS still showed lower *C. perfringens* spore germination than the negative control (*p* < 0.05) corresponding to a 2 log cycle reduction in viable cells. Overall, after up to 11 h of incubation, the formulations of the added PA-MD 1.50% and 2.50% (equivalent to 0.75% and 1.25% of PA, respectively) had a similar or higher performance when compared to the positive controls, 10% SS and 0.015% NO_2_. Between 18 and 57 h of incubation, the formulations with PA-MD 1.50% and 2.50% showed *C. perfringens* spores germination in the same range as 10% SS and 0.015% NO_2_ (*p* > 0.05). In general, 10% SS was the additive (positive control) that performed the best; however, it must be noted that it was added in very high amounts. On the contrary, NO_2_ was added to the sausages in very low amounts (0.015%), which may possibly explain the lower inhibition when compared with the other tested antimicrobials.

Table 1 shows the growth parameters that were predicted by the logistic model (LM) by the use of predictive microbiology. For all assays no lag phase (λ) was observed, which may be related to the inoculum adaptation, indicating that *C. perfringens* was adapted for rapid growth [44].

The negative control presented the highest maximum specific growth rate (μ) and the highest logarithmic increase in population (A) (Table 1), as expected due the absence of inhibitors. The second highest value of μ and A was observed in the formulation with the added 0.015% NO_2_. The values of μ in the formulations with PA-MD 1.50% and 2.50% were close to each other, and lower than the negative and positive control with NO_2_. The same behaviour of formulations with PA-MD 1.50% and 2.50% was observed for parameter A. The formulations containing PA in both proportions (0.75% and 1.25%) presented lower A values than the negative and positive control with NO_2_. The positive control containing SS 10% as an inhibitor was the formulation with the lowest μ and A (Table 1). In terms of adjustment, the logistic model presented a suitable adjustment for the observed and predicted values as evidenced by MSE, BF, AF, and R^2^.

**Figure 5:**
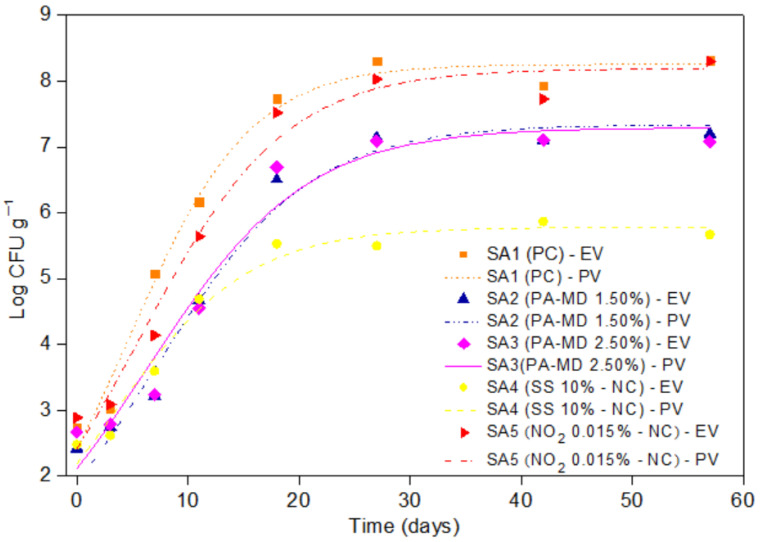
*Clostridium perfringens* growth curve in cooked sausage (SA) formulations that have been adjusted to the logistic model (LM). PA-MD: phytic acid-maltodextrin; SS: sodium sorbate; NO_2_: sodium nitrite; PC: positive control; NC: negative control; EV: experimental values; PV: predicted values by LM.

Figure 5 shows *C. perfringens* growth curve of sausage formulations that were adjusted to the logistic model, presenting a better visualization of the inhibitory effect of PA-MD, SS, and NO_2_ against *C. perfringens* spores on sausages.

The formulations with PA-MD 1.50% and 2.50% presented satisfactory inhibitory results compared with the negative control, and both demonstrated inhibitory results that were higher than those that were obtained with NO_2_, the inhibitor that is most widely used in the meat products industry. Compared to NO_2_, PA is a natural ingredient with great potential for application in the meat industry, both for its antimicrobial and antioxidant potential, especially with its chelating power [27,45]. In this sense, a new natural food preservative composed of PA and MD (1:1) is proposed for use in the range from 1.50% to 2.50% (*w/w*) in meat products.

## 4. Conclusions

In this work, the antimicrobial effect of phytic acid against spore germination and vegetative cell growth of *C. perfringens* type A was demonstrated and proven for the first time. The results showed that 1% PA was able to inhibit the germination of *C. perfringens* spores during 9.5 h of incubation, as well as having an inhibitory effect on the vegetative cell growth over 12 h of incubation. In vegetative cell growth, the inhibitory effect of 1% phytic acid was comparable to that which was obtained with 10% sodium sorbate. Additionally, an additive that is composed of phytic acid and maltodextrin (PA-MD) (1:1; *w/w*) that is applied in the proportion of 1.50% and 2.50%, showed satisfactory inhibitory effects against the germination of *C. perfringens* spores when evaluated in cooked sausage. In an unprecedented way, the present study demonstrated the possibility of phytic acid use as a new alternative food preservative in the meat products industry.

## 5. Patents

Canan, C.; Bloot, A.P.M.; Baraldi, I.J.; Colla, E.; Feltrin, V.P.; Corso, M.P.; Habu, S.; Nogues, D.R.N.; Kalschne, D.L.; Silva-Buzanello, R.A. Da Composição antimicrobiana a base de fitato, e seu uso 2018, BR 10 2018 006228 0, 1–34.

## Figures and Tables

**Table 1:** The growth curve parameters and statistical indices of the ML model adjustment to the experimental data of growth curves.

Formulations	λ	*A*	μ	MSE	BF	AF	R^2^
SA1 (NC)	0	8.26	0.38	0.069	1.001	1.057	0.99
SA2 (PA-MD 1.50%)	0	7.34	0.27	0.102	1.032	1.082	0.98
SA3 (PA-MD 2.50%)	0	7.29	0.26	0.161	1.011	1.092	0.98
SA4 (SS 10%-PC)	0	5.78	0.23	0.045	1.003	1.061	0.99
SA5 (NO_2_ 0.015%-PC)	0	8.19	0.31	0.109	1.008	1.069	0.99

λ: duration of the lag phase (h); μ: maximum specific growth rate (h^−1^); *A*: logarithmic increase in population (log CFU g^−1^); SA: cooked sausage; PA-MD: phytic acid-maltodextrin; SS: sodium sorbate; NO_2_: sodium nitrite; PC: positive control; NC: negative control; MSE: mean square error; BF: bias factor; AF: accuracy factor; R^2^: determination coefficient.

## Data Availability

The data presented in this study are available on request from the corresponding author.
